# Long non‐coding RNA AFAP1‐AS1/miR‐320a/RBPJ axis regulates laryngeal carcinoma cell stemness and chemoresistance

**DOI:** 10.1111/jcmm.13707

**Published:** 2018-07-04

**Authors:** Zhennan Yuan, Cheng Xiu, Kaibin Song, Rong Pei, Susheng Miao, Xionghui Mao, Ji Sun, Shenshan Jia

**Affiliations:** ^1^ Department of Head and Neck Surgery Harbin Medical University Cancer Hospital Harbin China

**Keywords:** AFAP1‐AS1, chemoresistance, laryngeal carcinoma, miR‐320a, RBPJ, stemness

## Abstract

AFAP1‐AS1 is a long non‐coding RNA that is associated with tumorigenesis and poor prognosis in a variety of cancers. We have been suggested that AFAP1‐AS1 increases tumorigenesis in laryngeal carcinoma specifically by enhancing stemness and chemoresistance. We assessed AFAP1‐AS1 expression in human laryngeal specimens, paired adjacent normal tissues and human HEp‐2 cells. Indeed, we found not only that AFAP1‐AS1 was up‐regulated in laryngeal carcinoma specimens and cells, but also that stemness‐associated genes were overexpressed. Silencing of AFAP1‐AS1 promoted HEp‐2 cell chemoresistance under cisplatin treatment. Expression of AFAP1‐AS1 was increased in drug‐resistant Hep‐2 cells. We then probed the mechanism of AFAP1‐AS1 activity and determined that miR‐320a was a potential molecular target of AFAP1‐AS1. Luciferase reporter and qRT‐PCR assays of AFAP1‐AS1 and miR‐320a levels in human specimens and cell cultures indicated that AFAP1‐AS1 negatively regulates miR‐320a. To discover the molecular mechanism of miR‐320a, we again used the DIANA Tools algorithm to predict its genetic target, RBPJ. After cloning the 3′‐untranslated regions (3′‐UTR) of RBPJ into a luciferase reporter, we determined that miR‐320a did in fact reduce RBPJ mRNA and protein levels. Ultimately, we determined that AFAP1‐AS1 increases RBPJ expression by negatively regulating miR‐320a and RBPJ overexpression rescues stemness and chemoresistance inhibited by AFAP1‐AS1 silencing. Taken together, these results suggest that AFAP1‐AS1 can serve as a prognostic biomarker in laryngeal carcinoma and that miR‐320a has the potential to improve standard therapeutic approaches to the disease, especially for cases in which cancer cell stemness and drug resistance present significant barriers to effective treatment.

## 1 INTRODUCTION

Laryngeal carcinoma is the most prevalent of head and neck cancers, accounting for about 25% to 30% of cases.^1^, ^2^ While there have been significant advances made in patient outcomes, these are largely due to improvements in prevention, such as reductions in cigarette smoking, and early diagnosis. Early stage laryngeal carcinomas (T1 and T2) can have up to an 80% to 90% cure rate, whereas advanced tumours (T3 or T4, N1‐N3 or M1) have only a 60% chance of cure.^3^, ^4^ It is therefore crucial to improve not just the methods of diagnosing laryngeal carcinoma, but also to advance treatment strategies for later stage disease. In the past few decades, the primary treatment approach evolved from total laryngectomy towards non‐surgical, organ‐preserving interventions with radiotherapy or chemoradiotherapy.^5^ Cisplatin is currently the chemotherapeutic standard and can be used in combination with taxane and 5‐fluorouracil, and when followed by radiotherapy can serve as an alternative to extensive surgery.^6^ However, treatment successes are severely limited by chemoresistance and cancer cell stemness, which both enhances chemoresistance and contributes to cancer cell proliferation in its own right.^7^, ^8^ In this study, we have identified the AFAP1‐AS1/miR‐320a/RBPJ axis as a regulator of laryngeal carcinoma cell stemness and chemoresistance.

It is well established that the prevalence of cancer stem cells (CSCs) and heightened propensity for tumours to maintain CSC subpopulations, also known as stemness, cause increased cancer aggressiveness and poorer patient outcomes.^9^, ^10^ In fact, it was recently discovered that reducing CD133+ CSC populations in laryngeal carcinoma cell cultures can heighten the effectiveness of cisplatin treatment.^11^ These results were due not only to the impact of reducing CSCs on cancer cell longevity, but also to the substantial effects that CSCs have on enhancing chemoresistance.^12^, ^13^ It is likely that inherent cancer cell drug resistance arises from the natural tendency of CSCs for heightened DNA repair, cell quiescence and the expression of ATP‐binding cassette transporters that promote drug efflux.^14^, ^15^ When CSCs survive an initial course of chemotherapy, they can adopt a drug resistance phenotype that contributes to acquired chemoresistance.^16^ In laryngeal carcinoma, a number of mechanisms of chemoresistance have been identified,^17^, ^18^ including the dysregulation of microRNAs (miRNAs) that regulate gene expression.^19^ In this study, we sought to identify a novel molecular mechanism of laryngeal carcinoma stemness and chemoresistance.

Actin filament‐associated protein 1 antisense RNA1 (AFAP1‐AS1) is a long non‐coding RNA (lncRNA) derived from the antisense strand of DNA at the *AFAP1* coding gene locus. It has been associated with several cancer types, especially head and neck squamous cell carcinomas (HNSCCs). lncRNAs are RNA transcripts longer than 200 nucleotides but that lack significant open‐reading frames.^20^ While not ultimately translated into proteins, lncRNAs participate in numerous physiological activities, including chromosome modification, transcriptional activation and interference, and cell growth, differentiation and apoptosis.^21^, ^22^ Apart from their role in cellular physiology, lncRNAs, especially when dysregulated, can contribute to oncogenesis.^23^, ^24^ In 2013, Wu et al^25^ determined that AFAP1‐AS1 overexpression promotes oncogenesis in Barrett's esophagus (BE) and oesophageal adenocarcinoma. AFAP1‐AS1 has also been implicated in a number of other cancers, including hepatocellular carcinoma,^26^ lung cancer^27^ and nasopharyngeal carcinoma.^28^ In this study, we have been suggested that AFAP1‐AS1 promotes oncogenesis in laryngeal carcinoma by enhancing cancer cell stemness and chemoresistance. Ultimately, we found not only that AFAP1‐AS1 increases laryngeal carcinoma stemness and chemoresistance, but also that it does so by regulating miR‐320a activity and RBPJ expression. This study therefore provides the basis for developing biomarkers and treatment strategies with the potential to dramatically improve patient outcomes.

## 2 MATERIALS AND METHODS

### 2.1 Patient specimens

A total of 24 human laryngeal specimens and paired adjacent normal tissues were obtained from the Harbin Medical University Cancer Hospital. Prior to operation, patients did not receive chemo‐ or radiotherapy. All laryngeal specimens and normal tissues were snap‐frozen in liquid nitrogen immediately after surgery and stored in liquid nitrogen for further analyses. Histological diagnoses were classified by three pathologists. Before surgery at the centre, all patients provided written informed consent to allow for any excess tissue to be used for research studies.

### 2.2 Cell culture and transfection

We obtained human epithelial type 2 (HEp‐2) cells from American Type Culture Collection (ATCC, Manassas, VA, USA) and cultured them in Dulbecco's modified Eagle's Medium (DMEM) supplemented with 10% foetal bovine serum (FBS), 100 U/mL penicillin and 0.1 mg/mL streptomycin under humidified conditions of 95% air and 5% CO_2_ at 37°C. For tumour sphere cultures, HEp‐2 cells were maintained in DMEM/F‐12 medium containing 2% B27 (Invitrogen, Carlsbad, CA, USA), 1% N2 (Invitrogen), 20 ng/mL epidermal growth factor (EGF, Invitrogen), 20 ng/mL basic fibroblast growth factor (bFGF, Invitrogen) and penicillin/streptomycin. For cisplatin‐resistant HEp‐2 generations, HEp‐2 cells were cultured in growing medium containing cisplatin with gradually increasing concentration (0.5, 1, 1.5 and 2 μmol L^−1^). Cells were maintained for three months under each cisplatin concentration. Transfection protocol followed the Lipofectamine™ 3000 (Invitrogen) transfection reagent instructions.

### 2.3 RNA extraction and quantitative real‐time PCR (qRT‐PCR)

For clinical samples and cultured cell lines, total RNA was purified using the TRIzol kit (Tiangen Biotech, Beijing, China) according to the manufacturer's protocols. Primers for reverse transcription and PCR were generated by Ribo Biotech (Guangzhou, Guangdong, China). Expression levels were quantified by qRT‐PCR with the SYBR Premix Ex Taq Kit (Takara, Dalian, Liaoning, China). qRT‐PCR was performed in a DNA Engine Opticon2 system (Bio‐Rad, Richmond, CA, USA). The following PCR protocol was used: denaturation at 95°C for 3 minutes, followed by amplification for 40 cycles at 95°C for 12 seconds and at 62°C for 40 seconds. The melting curve was plotted from 62 to 95°C and read every 0.2°C with a 2 seconds hold. GAPDH and U6 small nuclear RNA were used as internal controls. The results were represented as fold changes, which were calculated by the 2^−ΔΔCT^ method.

### 2.4 Tumour sphere formation

HEp‐2 cells (1 × 10^4^/well) were seeded in low‐attachment six‐well plates (Corning, Corning, NY, USA) and cultured for 1 week in modified DMEM/F‐12 medium containing 2% B27, 1% N2, 20 ng/mL EGF, 20 ng/mL bFGF and penicillin/streptomycin. Medium was changed every 2 days.

### 2.5 Luciferase assay

4.0 × 10^4^ HEp‐2 cells were cotransfected with 200 ng of miRNA mimics, 200 ng of the indicated pGL3 firefly luciferase construct and 20 ng of a pGL3 Renilla luciferase construct as normalization control. The medium was changed 6 hour post‐transfection, and luciferase activity was measured after 48 hour using the dual‐luciferase reporter assay system (Promega, Madison, WI, USA).

### 2.6 Western blot

HEp‐2 cells were harvested in RIPA buffer with protease inhibitor (Beyotime, Beijing, China). Lysates were centrifuged, and protein was quantified using the BCA assay kit (Beyotime). Lysates were resolved by electrophoresis, transferred to a poly‐vinylidene difluoride membrane (Millipore, Bedford, MA, USA) and probed with primary antibodies against RBPJ or GAPDH (Abcam, Cambridge, Massachusetts, USA) overnight at 4°C. The antibodies were diluted 1:1000. After secondary antibody horseradish peroxidase (HRP) conjugation, the enhanced chemiluminescence detection kit (Beyotime) was used for signal detection.

### 2.7 Cell viability

HEp‐2 cells were seeded in a 96‐well plate at a density of 3000 cells per well. Cells were incubated with 10% CCK8 reagent (DoJinDo Laboratories, Japan) for 1 hour at 37°C. Plates were then analysed using an automatic spectrometer (Multimode Reader; EnSpire) at 450 nm.

### 2.8 Cell DAPI staining

We conducted nuclear DAPI staining to access cell apoptosis using Cell Apoptosis DAPI Detection Kit (GenScript, Piscataway, NJ) according to manufacturer's instructions.

### 2.9 Statistical analysis

All of the results are expressed as the means and totals derived from independent experiments. When comparing two groups, Student's unpaired *t* test (two‐tailed) was used. For all tests, a *P* value <.05 was considered significant. The Benjamini and Hochberg false discovery rate was used as a correction for multiple testing. Error bars represent the SDs of at least three independent experiments.

## 3 RESULTS

### 3.1 The expression of AFAP1‐AS1 is up‐regulated in laryngeal carcinoma specimens and promotes laryngeal carcinoma cell stemness

**Figure 1:**
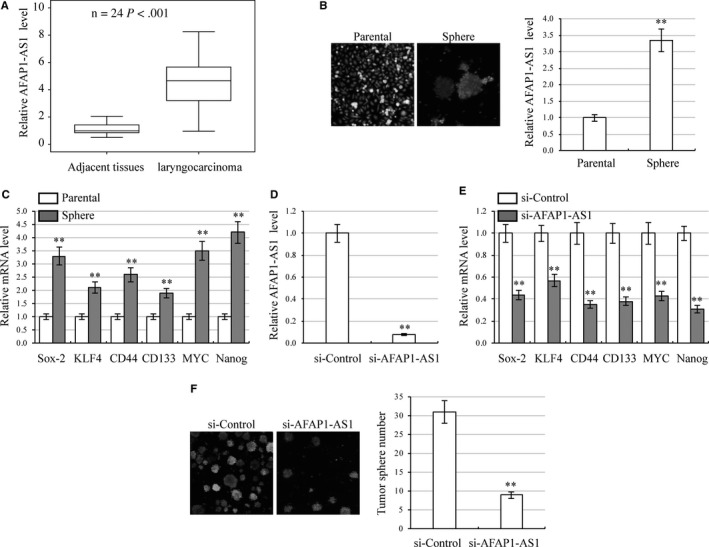
The expression of AFAP1‐AS1 is up‐regulated in laryngeal carcinoma specimens and promotes laryngeal carcinoma cell stemness. A, Elevated expression of AFAP1‐AS1 in 24 laryngeal carcinoma specimens compared with adjacent normal tissues by qRT‐PCR. *P *<* *.001. B, HEp‐2 cell morphology of parental cells and stemness‐enriched cell spheres (left) and corresponding AFAP1‐AS1 expression by qRT‐PCR (right). **P <* *.01, compared with parental cells. C, Expression of stemness‐associated genes (*Sox‐2*,* KLF4*,* CD44*,* CD133*,* MYC* and *Nanog*) in parental cells and stemness‐enriched cell spheres. Gene expression was analysed by qRT‐PCR. D, HEp‐2 cells were transfected with AFAP1‐AS1 siRNA to inhibit AFAP1‐AS1 expression. AFAP1‐AS1 levels were analysed by qRT‐PCR. **P <* *.01, compared with control siRNA transfected cells. E, Expression of stemness‐associated genes in AFAP1‐AS1 silenced HEp‐2 cells. Gene expression was analysed by qRT‐PCR. **P <* *.01, compared with control siRNA transfected cells. F, Number of tumour spheres in AFAP1‐AS1 silenced HEp‐2 cells. **P <* *.01, compared with control siRNA transfected cells

To determine whether AFAP1‐AS1 contributes to the tumorigenesis of laryngeal carcinoma, we used qRT‐PCR to assess AFAP1‐AS1 expression levels in human laryngeal carcinoma specimens and adjacent normal paracarcinoma tissues. AFAP1‐AS1 levels in laryngeal carcinoma specimens were significantly decreased compared with those in paired normal tissues (24 pairs, *P *<* *.001) (Figure 1A). To explore the role of AFAP1‐AS1 in laryngeal carcinoma cell stemness, we performed qRT‐PCR to detect AFAP1‐AS1 expression between parental cells and stemness‐enriched cell spheres. AFAP1‐AS1 was significantly increased in cell spheres (Figure 1B). Higher expression of stemness‐associated genes in HEp‐2 cell spheres than in parental cells was confirmed by qRT‐PCR (Figure 1C). These genes were used as markers to indicate the stemness of CSC.^29^, ^30^, ^31^ To further explore AFAP1‐AS1 function in laryngeal carcinoma cell development, we knocked down AFAP1‐AS1 expression using siRNA transfection and confirmed silencing with qRT‐PCR (Figure 1D). We then used qRT‐PCR to demonstrate that stemness‐associated gene expression in the AFAP1‐AS1‐silenced cells was significantly reduced compared with control cells (Figure 1E). Using tumour sphere assays, we then demonstrated that AFAP1‐AS1 moderately inhibited cell self‐renewal (Figure 1F). Overall, these results suggest that AFAP1‐AS1 contributes to laryngeal carcinoma tumorigenesis by promoting cancer cell stemness.

### 3.2 AFAP1‐AS1 enhances cisplatin resistance in laryngeal carcinoma cells

**Figure 2:**
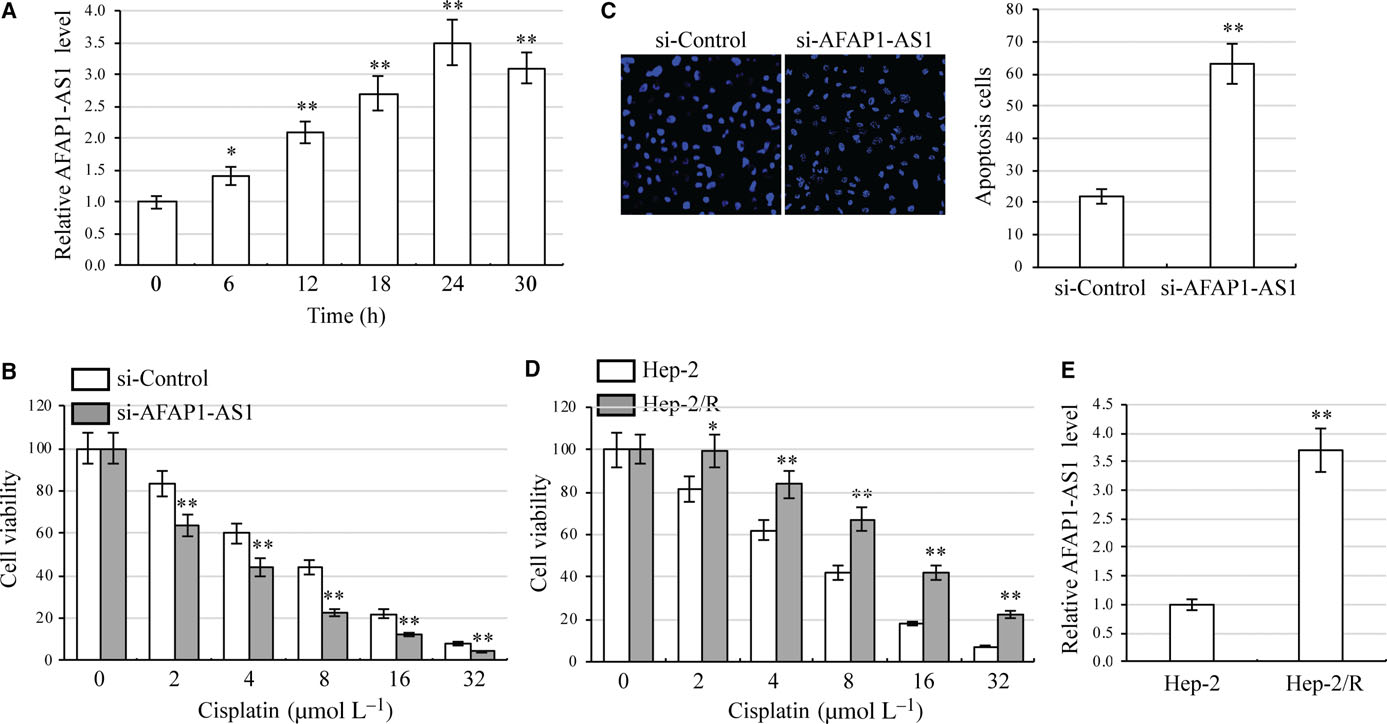
AFAP1‐AS1 enhances cisplatin resistance in laryngeal carcinoma cells. A, HEp‐2 was treated with 4 μmol L^−1^ cisplatin. Expression of AFAP1‐AS1 was analysed at various times (0, 6, 12, 18, 24, and 30 h) by qRT‐PCR. *P <* *.05, **P <* *.01, compared with 0 h. B, AFAP1‐AS1 silenced HEp‐2 cells were cultured in 96‐well plates. Cell viability was analysed by CCK8 assay under treatment with various concentration of cisplatin (0, 2, 4, 8, 16 and 32 μmol L^−1^). **P <* *.01, compared with control siRNA transfected cells. C, Apoptosis assays in AFAP1‐AS1 silenced HEp‐2 cells under 8 μmol L^−1^ cisplatin treatment. **P <* *.01, compared with control siRNA transfected cells. D, Cisplatin‐resistant HEp‐2 cell lines (HEp‐2/R) were established. Cell viability assays were performed in HEp‐2 and HEp‐2/R cells under various concentrations of cisplatin treatment. *P <* *.05, **P <* *.01, compared with HEp‐2 cells. E, Expression of AFAP1‐AS1 in HEp‐2 and HEp‐2/R cells was analysed by qRT‐PCR. **P <* *.01, compared with HEp‐2 cells

Chemoresistance is an essential characteristic of CSC.^32^ To investigate whether AFAP1‐AS1 functions as a dominant determinant of chemoresistance in laryngeal carcinoma cells, we treated AFAP1‐AS1‐silenced HEp‐2 cells with 4 μmol L^−1^ cisplatin and analysed AFAP1‐AS1 expression at different time‐points. AFAP1‐AS1 expression was increased in a time‐dependent manner and reached peak expression 24 hour after treatment (Figure 2A). AFAP1‐AS1‐silenced HEp‐2 cells were then treated with various concentration of cisplatin. CCK8 assays of these cells demonstrated significant inhibition of HEp‐2 cell viability (Figure 2B). Apoptosis assays of AFAP1‐AS1‐silenced HEp‐2 cells treated with 8 μmol L^−1^ cisplatin showed that silencing AFAP1‐AS1 significantly elevated cell apoptosis (Figure 2C). The nucleus in apoptotic cells showed sign of blebbing or fragmentation. We then generated cisplatin‐resistant HEp‐2 (HEp‐2/R) cells and confirmed their resistance using cell viability assays (Figure 2D). Elevated expression of AFAP1‐AS1 was found in HEp‐2/R cells in contrast to HEp‐2 cells (Figure 2E).

### 3.3 AFAP1‐AS1 targets miR‐320a

**Figure 3:**
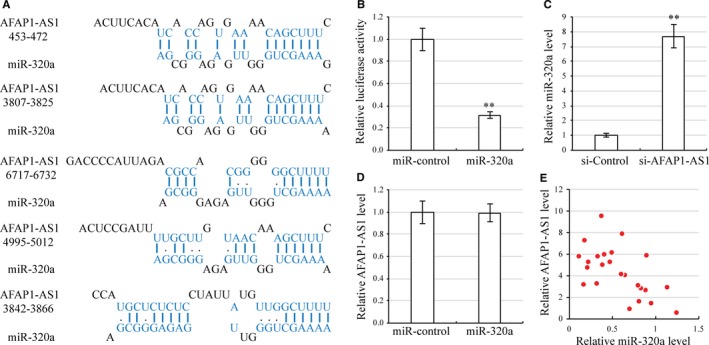
AFAP1‐AS1 targets miR‐320a. A, Sequence of the miR‐320a binding sites within the human AFAP1‐AS1 labelled in blue. B, Luciferase assay in HEp‐2 cells, which were cotransfected with miR‐Control or miR‐320a and a luciferase reporter containing the full length AFAP1‐AS1. **P <* *.01, compared with control miRNA transfected cells. C, Expression of miR‐320a in AFAP1‐AS1 silenced HEp‐2 cells. **P <* *.01, compared with control siRNA transfected cells by qRT‐PCR. D, Expression of AFAP1‐AS1 in miR‐320a overexpression HEp‐2 cells by qRT‐PCR. E, Spearman's correlation analysis was used to determine the correlation between AFAP1‐AS1 and miR‐320a expression in human laryngeal carcinoma specimens. *r *=* *−5.9

To discover the molecular mechanism of AFAP1‐AS1, we used the DIANA Tools (http://www.microrna.gr) miRNA target prediction algorithm.^33^, ^34^ miR‐320a was predicted to bind with AFAP1‐AS1 at multiple sites (Figure 3A). To verify whether miR‐320a is a genetic target of AFAP1‐AS1, we cloned the full length AFAP1‐AS1 into a luciferase reporter. Luciferase activity decreased by 64% when miR‐320a was overexpressed as compared with control miRNA (miR‐Control) (Figure 3B). Furthermore, we found that AFAP1‐AS1 down‐regulation in HEp‐2 cells caused the endogenous level of miR‐320a to increase significantly (Figure 3C). However, overexpression of miR‐320a did not alter AFAP1‐AS1 levels (Figure 3D). We then performed qRT‐PCR to analyse the correlation between AFAP1‐AS1 and miR‐320a levels in 24 laryngeal carcinoma specimens. The endogenous expression of AFAP1‐AS1 was negatively associated with the expression of the miR‐320a (Figure 3E). The totality of these findings suggests that AFAP1‐AS1 negatively regulates miR‐320a activity.

### 3.4 miR‐320a reduces stemness and cisplatin resistance in laryngeal carcinoma cells

**Figure 4:**
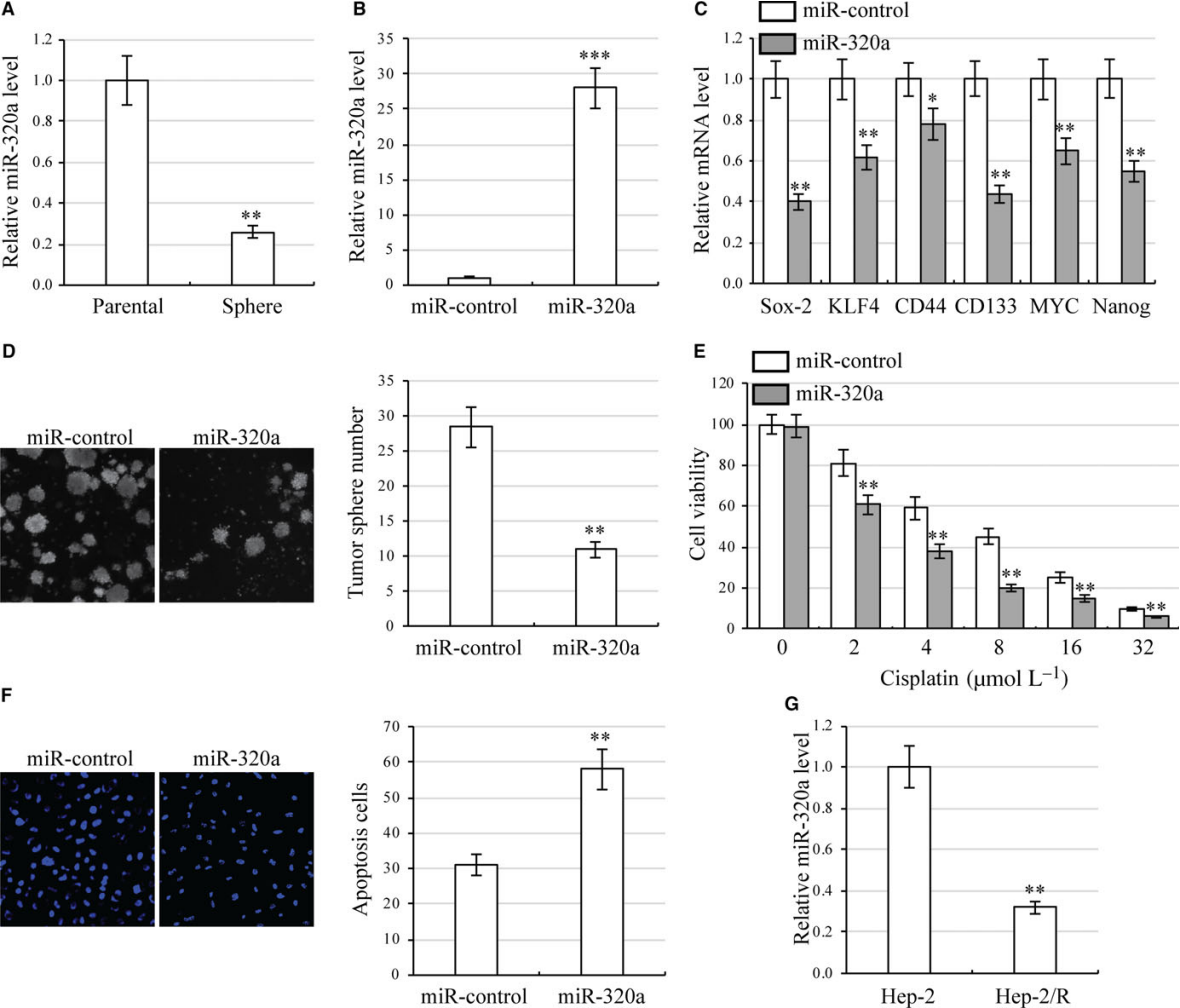
miR‐320a reduces stemness and cisplatin resistance in laryngeal carcinoma cells. A, HEp‐2 cell morphology of parental cells and stemness‐enriched cell spheres (left) and corresponding miR‐320a expression (right). **P <* *.01, compared with parental cells. B, Expression of miR‐320a in miR‐320 overexpression HEp‐2 cells by qRT‐PCR. ***P <* *.001, compared with control miRNA transfected cells. C, Expression of stemness‐associated genes in miR‐320a overexpression HEp‐2 cells. Gene expression was analysed by qRT‐PCR. *P <* *.05, **P <* *.01, compared with control miRNA transfected cells. D, Number of tumour spheres in miR‐320a overexpression HEp‐2 cells. **P <* *.01 compared with control miRNA transfected cells. E, miR‐320a overexpression HEp‐2 cells were cultured in 96‐well plates. Cell viability was analysed using CCK8 assays under various concentrations of cisplatin (0, 2, 4, 8, 16 and 32 μmol L^−1^). **P <* *.01, compared with control miRNA transfected cells. F, Apoptosis assay in miR‐320a overexpression HEp‐2 cells under 8 μmol L^−1^ cisplatin treatment. **P <* *.01, compared with control miRNA transfected cells. G, Expression of miR‐320a in HEp‐2 and HEp‐2/R cells was analysed by qRT‐PCR. **P <* *.01, compared with HEp‐2 cells

To explore the possible role of miR‐320a in laryngeal carcinoma cell stemness, we performed qRT‐PCR to detect miR‐320a expression between parental cells and stemness‐enriched cell spheres. We found that miR‐320a was down‐regulated in cell spheres (Figure 4A). To further explore miR‐320a function in laryngeal carcinoma cell development, we increased miR‐320a expression by miR‐320a transfection, confirming miR‐320a overexpression by qRT‐PCR (Figure 4B). qRT‐PCR of these cells then revealed that expression levels of stemness‐associated genes were significantly reduced in miR‐320a overexpression cells as compared to miR‐control cells (Figure 4C). Tumour sphere assays then showed that AFAP1‐AS1 overexpression moderately inhibited cell self‐renewal (Figure 4D). To investigate whether miR‐320a regulates laryngeal carcinoma cell cisplatin resistance, miR‐320a overexpression HEp‐2 cells were treated with various concentration of cisplatin. CCK8 assays demonstrated that miR‐320a overexpression significantly inhibited HEp‐2 cell viability (Figure 4E). Apoptosis assays then revealed that miR‐320a overexpression in cisplatin‐treated HEp‐2 cells significantly elevated apoptosis (Figure 4F). Furthermore, decreased expression of AFAP1‐A miR‐320a was found in HEp‐2/R cells in contrast to HEp‐2 cells (Figure 4G). These results indicate that miR‐320a overexpression reduces laryngeal carcinoma cell stemness while increasing chemosensitivity to cisplatin.

### 3.5 miR‐320a targets RBPJ

**Figure 5:**
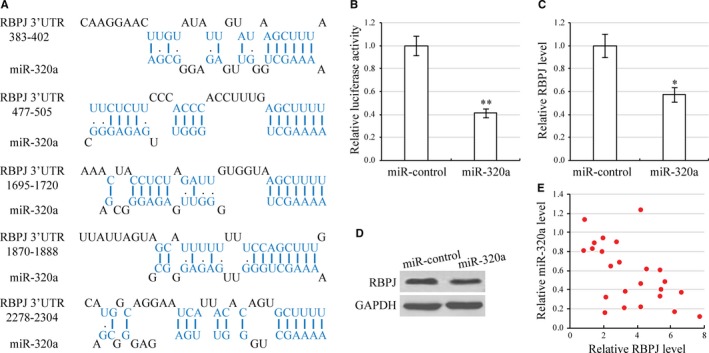
miR‐320a targets RBPJ. A, Sequence of the miR‐320a binding sites within the human RBPJ 3′‐UTR. B, Luciferase assay in HEp‐2 cells, which were cotransfected with miR‐Control or miR‐320a and a luciferase reporter containing the full length RBPJ 3′‐UTR. C, Expression of RBPJ mRNA in miR‐320 overexpression HEp‐2 cells by qRT‐PCR. *P <* *.05, compared with control miRNA transfected cells. D, Expression of RBPJ protein in miR‐320 overexpression HEp‐2 cells by Western blot. **P <* *.01, compared with control miRNA transfected cells. E, Spearman's correlation analysis was used to determine the correlation between miR‐320a and RBPJ expression levels in human laryngeal carcinoma specimens. *r *=* *−5.6

To discover the molecular mechanism of miR‐320a, we used the DIANA Tools algorithm to predict its target gene, *RBPJ* (Figure 5A). To verify whether *RBPJ* was a target gene of miR‐320a, we cloned the 3′‐untranslated regions (UTR) of RBPJ into a luciferase reporter. The luciferase activity of the RBPJ 3′‐UTR decreased by 60% when miR‐320a was overexpressed as compared with control (Figure 5B). Furthermore, we found that miR‐320a overexpression in HEp‐2 cells reduced both mRNA and protein levels of RBPJ (Figure 5C,D). qRT‐PCR of 24 human laryngeal carcinoma specimens revealed that endogenous expression of miR‐320a was negatively associated with the expression of the RBPJ (Figure 5E). Overall, these experiments highly suggest that miR‐320a negatively regulates *RBPJ* expression.

### 3.6 AFAP1‐AS1 regulates laryngeal carcinoma cells through miR‐320a/RBPJ

**Figure 6:**
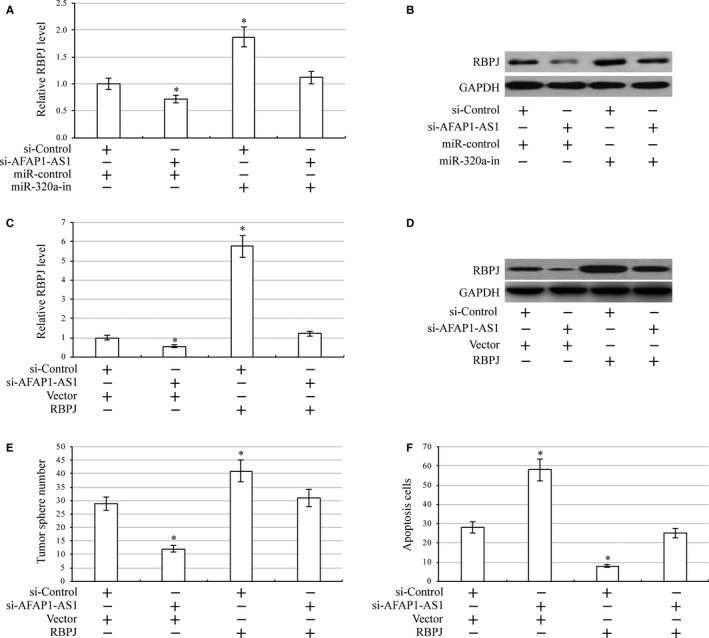
AFAP1‐AS1 regulates laryngeal carcinoma cells through miR‐320a/ RBPJ. A, Expression of RBPJ mRNA in AFAP1‐AS1 silenced, miR‐320a inhibition (miR‐320a‐in) and AFAP1‐AS1 silenced plus miR‐320a inhibition HEp‐2 cells by qRT‐PCR. *P <* *.05, compared with control cells. B, Expression of RBPJ protein in AFAP1‐AS1 silenced, miR‐320a inhibition and AFAP1‐AS1 silenced plus miR‐320a inhibition HEp‐2 cells by Western blot. C, Expression of RBPJ mRNA in AFAP1‐AS1 silenced, RBPJ and AFAP1‐AS1 silenced plus RBPJ HEp‐2 cells by qRT‐PCR. *P <* *.05, compared with control cells. D, Expression of RBPJ mRNA in AFAP1‐AS1 silenced, RBPJ and AFAP1‐AS1 silenced plus RBPJ HEp‐2 cells by Western blot. E, Number of tumour spheres in AFAP1‐AS1 silenced, RBPJ and AFAP1‐AS1 silenced plus RBPJ HEp‐2 cells. *P <* *.05 compared with control cells. F, Apoptosis assay in AFAP1‐AS1 silenced, RBPJ and AFAP1‐AS1 silenced plus RBPJ HEp‐2 cells under 8 μmol L^−1^ cisplatin treatment. *P <* *.05, compared with control cells

To investigate whether AFAP1‐AS1 regulates *RBPJ* expression, we analysed mRNA and protein levels of RBPJ in AFAP1‐AS1 silenced HEp‐2 cells. Indeed, when AFAP1‐AS1 was silenced, *RBPJ* expression was significantly inhibited (Figure 6A,B). As expected, inhibition of miR‐320a increased RBPJ mRNA and protein levels. In addition, miR‐320a inhibition prevented AFAP1‐AS1 silencing that had previously caused *RBPJ* down‐regulation (Figure 6A,B). Furthermore, we investigated the role of RBPJ in laryngeal carcinoma cell development. We found down‐regulation of RBPJ significantly reduced HEp‐2 cell stemness and chemoresistance (Supporting information Figure S1). To analyse whether AFAP1‐AS1 regulates laryngeal carcinoma cells through miR‐320a‐mediated RBPJ, we increased RBPJ expression in AFAP1‐AS1 silencing HEp‐2 cells (Figure 6C,D). As we expected, overexpression of RBPJ rescued tumour sphere number and drug‐induced apoptosis of laryngeal carcinoma cells (Figure 6E,F). These results determine convincingly that AFAP1‐AS1 regulates laryngeal carcinoma cells through miR‐320a/RBPJ.

## 4 DISCUSSION

In agreement with the role of AFAP1‐AS1 as a promoter of oncogenesis in a variety of cancers, including head and neck squamous cell carcinoma (HNSCC),^28^ our findings support the hypothesis that AFAP1‐AS1 contributes to laryngeal carcinoma cell stemness and chemoresistance. Using human tumour specimens and paired adjacent normal tissues from 24 patients, as well as the HEp‐2 cell line, we demonstrated with qRT‐PCR, small interfering RNAs (siRNAs) and tumour sphere assays that AFAP1‐AS1 is significantly up‐regulated in laryngeal cancer cells. After determining that AFAP1‐AS1 promotes cancer cell stemness and cisplatin chemoresistance, we used the DIANA Tools gene targeting prediction algorithm, luciferase reporter assays and CCK8 cell viability assays to demonstrate that miR‐320a overexpression reduces laryngeal carcinoma cell stemness while increasing chemosensitivity to cisplatin. The further use of DIANA Tools and a number of cell biology assays allowed us to determine conclusively that miR‐320a targets RBPJ and that AFAP1‐AS1 increases *RBPJ* expression by negatively regulating miR‐320a, thereby giving rise to laryngeal carcinoma's stemness and chemoresistance.

MicroRNAs (miRNAs) are short (20‐24 nucleotides), non‐coding, evolutionarily conserved RNAs that regulate gene expression post‐transcriptionally by binding the 3′ untranslated regions (3′‐UTRs) of target mRNAs.^35^ Their activity is crucial for a wide range of necessary physiological processes such as cell development, cell proliferation, gene regulation and apoptosis.^36^ However, miRNAs have also been implicated in a number of pathologies, including a variety of cancers, and are therefore being recognized as potentially crucial biomarkers and treatment targets.^37^, ^38^ In fact, a number of studies have already established a role for miRNAs in laryngeal carcinoma diagnosis, prognosis and treatment. In 2013, Saito et al^39^ stratified human laryngeal carcinoma specimens according to malignancy and determined that miR‐196a was a promising biomarker and treatment target. Similarly, in 2014, Wang et al^40^ discovered that serum exosomal miR‐21 and HOTAIR in laryngeal carcinoma patients can serve as prognostic biomarkers. Even though the present study began with a hypothesis regarding the activity of AFAP1‐AS1 in laryngeal carcinoma, we made a crucial early discovery that miR‐320a is implicated in its effects on oncogenesis.

MiR‐320a has been identified in a number of studies largely as an inhibitor of oncogenesis, including cell proliferation, metastasis and chemoresistance. In 2017, Lv et al^41^ demonstrated that miR‐320a suppressed lung adenocarcinoma cell proliferation and metastasis and enhanced irradiation‐induced apoptosis by regulating signal transduced and activator of transcription 3. Furthermore, in 2014, Gao et al^42^ screened differentially expressed miRNAs in primary gastrointestinal stromal tumour patients and imatinib‐resistant patients, finding that miR‐320a down‐regulation was highly associated with chemoresistance. While there is an impressive amount of research into the role of miR‐320a in various cancers and its activity through a wide range of molecular mechanisms, such as the Wnt/beta‐catenin^43^ and vascular endothelial growth factor pathways,^44^ the present study is the first to establish its activity as a negative regulator of laryngeal carcinoma stemness and chemoresistance. It is also the first to demonstrate its regulation of *RBPJ* expression.

RBPJ plays a crucial role in the notch signalling pathway, including as a primary transcriptional effector and potentially as a maintainer of gene expression programs.^45^, ^46^ When associated with Notch proteins, RBPJ acts as a transcriptional activator, but when not associated with Notch proteins, it exists in complexes with corepressors and serves to block transcription.^47^ Even though there are no studies that link RBPJ activity to laryngeal carcinoma, in 2016, Liu et al^48^ found that somatic mutations in NOTCH1 correlated strongly with higher rates of recurrence and lower survival in HNSCC. It is likely that genes that regulate Notch signalling, such as *RBPJ*, are in some way responsible for altering HNSCC prognosis. In this study, we have determined for the first time that RBPJ is crucial for the regulation of laryngeal carcinoma cell stemness and chemoresistance. However, it is important that future studies strive to elucidate its exact mechanism of action. Overall, this study has laid an impressive groundwork for the discovery of clinically relevant biomarkers, treatment targets and strategies to improve chemotherapeutic effectiveness in laryngeal carcinoma.

## CONFLICT OF INTEREST

The authors declare that they have no conflict of interests.
