# Supplementary figures and images for: Long non‐coding RNA AFAP1‐AS1/miR‐320a/RBPJ axis regulates laryngeal carcinoma cell stemness and chemoresistance
Source: J Cell Mol Med. 2018 Jul 4;22(9):4253–62. doi: 10.1111/jcmm.13707 (PMC6111816; doi:10.1111/jcmm.13707)

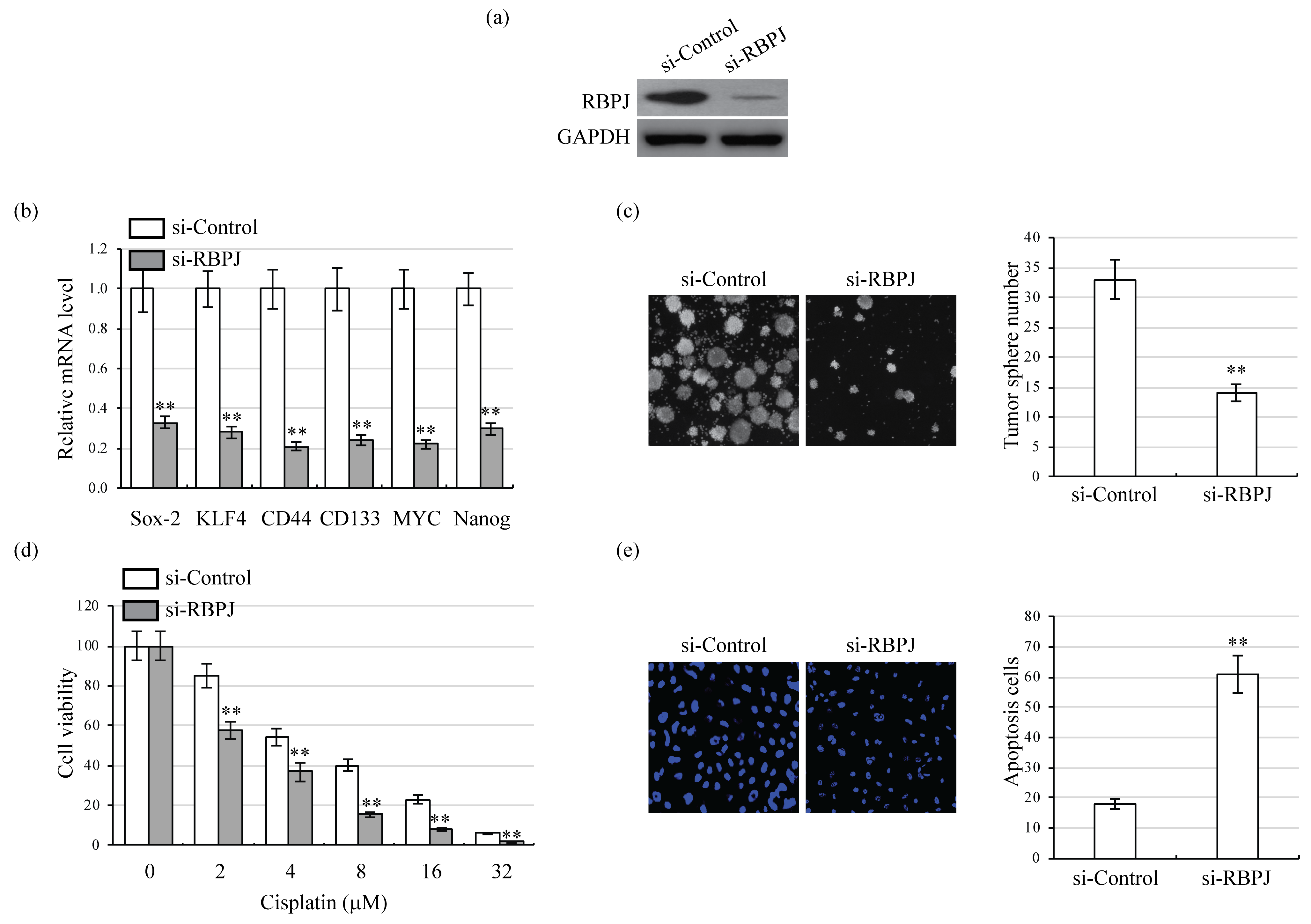

Supplement: Supplementary file 1 [file JCMM-22-4253-s001.tif]
