# Supplementary material for: Long non‐coding RNA AFAP1‐AS1/miR‐320a/RBPJ axis regulates laryngeal carcinoma cell stemness and chemoresistance
Source: J Cell Mol Med. 2018 Jul 4;22(9):4253–62. doi: 10.1111/jcmm.13707 (PMC6111816; doi:10.1111/jcmm.13707)
Supplement: Supplementary file 2 [file JCMM-22-4253-s002.docx]

**Figure S1** Knockdown of RBPJ decreases stemness and cisplatin resistance in laryngeal carcinoma cells. (a) Expression of RBPJ in RBPJ knockdown HEp-2 cells by western blot. (b) Expression of stemness-associated genes in RBPJ knockdown HEp-2 cells. Gene expression was analysed by qRT-PCR. ***p*<0.01, compared with control siRNA transfected cells. (c) Number of tumor spheres in RBPJ knockdown HEp-2 cells. ***p*<0.01 compared with control siRNA transfected cells. (d) RBPJ knockdown HEp-2 cells were cultured in 96-well plates. Cell viability was analysed using CCK8 assays under various concentrations of cisplatin (0, 2, 4, 8, 16, and 32 μM). ***p*<0.01, compared with control siRNA transfected cells. (e) Apoptosis assay in RBPJ knockdown HEp-2 cells under 8 μM cisplatin treatment. ***p*<0.01, compared with control siRNA transfected cells.
